# A systems approach to scale-up for population health improvement

**DOI:** 10.1186/s12961-021-00679-0

**Published:** 2021-03-01

**Authors:** Harriet Koorts, Harry Rutter

**Affiliations:** 1 School of Exercise and Nutrition Sciences, Institute for Physical Activity and Nutrition (IPAN), Deakin University, 221 Burwood Highway, Burwood, VIC 3125 Australia; 2 Department of Social and Policy Sciences, University of Bath, Claverton Down, Bath, United Kingdom

**Keywords:** Systems, Scale-up, Public health, Global health, Implementation

## Abstract

Despite a number of important global public health successes, for many health behaviours there is a continued lack of interventions that have been sufficiently scaled up to achieve system-wide integration. This has limited sustainable and equitable population health improvement. Systems change plays a major role in the relation between implementation processes and at-scale institutionalisation of public health interventions. However, in research, systems approaches remain underutilised in scaling up. Public health scale-up models have typically centred on intervention replication through linear expansion. In this paper, we discuss current conceptualisations and approaches used when scaling up in public health, and propose a new perspective on scaling that shifts attention away from the intervention to focus instead on achieving the desired population-level health outcomes. In our view, ‘scaling up’ exists on a continuum. At one end, effective scaling can involve a linear, intervention-orientated expansive approach that prioritises the spread of evidence-based interventions into existing systems in order to drive expansion in the application of that intervention. At the other end, we contend that scale-up can sit within a complex systems paradigm in which interventions are conceptualised as events in systems. In this case, implementation and scale-up activities should focus on generating changes within the system itself to achieve the desired outcome. This we refer to as ‘systems-orientated scale-up’ to achieving population health improvement, which can complement traditional approaches in relevant situations. We argue that for some health behaviours, our proposed approach towards scaling up could enhance intervention implementation, sustainability and population health impact.

## Main text

Scaling up actions to improve population health has been a focus of the WHO for over a decade [1]. In 2018, the WHO Independent High-level Commission on Noncommunicable Diseases (NCDs) stated a global priority to achieve ‘increasing investment in and implementation of evidence-based solutions [to NCDs] on a dramatically larger scale’ [2]. *Scale-up* has been defined as ‘replicating and extending the reach of an intervention into other localities, cities, or regions’ [3], in order to achieve sustainable health benefits. Despite a number of important public health successes, such as tobacco control measures which have reduced global smoking rates from 27% to 20% (2010–16) [4], for many health behaviours there is a continued lack of public health interventions that have been scaled up sufficiently to achieve sustainable and equitable population health improvement. There are multiple reasons why public health interventions might fail to achieve lasting population-wide impact, ranging from a lack of political prioritisation, and consequent lack of resources, to insufficient preparation to meet the challenges of implementing interventions in practice [5]. Another reason is that for some health behaviours, current scale-up approaches may be ineffective at achieving system-wide impact. In this paper, we discuss current conceptualisations and approaches used when scaling up in public health, and propose a new perspective on scaling that shifts attention away from the intervention to focus instead on achieving the desired population-level health outcomes. We argue that for some health behaviours, our proposed approach towards scaling up could enhance both sustainability and population health impact.

Greenhalgh and Papoutsi have conceptualised the spread and scale of interventions under three categories: implementation science, social science and complexity science [6]. Each of these provides a distinct change logic to inform and interpret scaling up action: implementation science typically promotes a sequential and mechanistic spread of interventions; social science tends to emphasize the mechanisms underpinning scale-up, i.e., to understand what works, for whom, and under what circumstances; and complexity science embraces the impact of complex systems when scaling, and the important interdependencies that can exist between systems, such as maintaining momentum within political systems in tandem with strengthening health system capacity [6]. Whilst complexity science and systems methods increasingly inform the expansion of programs [7], and the influence of system characteristics when scaling has unquestionably been recognised [8] (e.g., the “innovation-system fit” which is a critical feature of a system’s preparedness to accept change brought about by an intervention) [9]; implementation science approaches have dominated the scale-up literature. The majority of emphasis has been on the linear replication and expansion of interventions *into* existing systems [7]. Scale-up also typically begins with small-scale trials that move through to implementation in larger real-world settings, thus often mirroring the ‘pipeline model’ of research translation from efficacy to effectiveness and then scale-up [5].

We refer to this as ‘intervention-orientated scale-up’ in public health, which we define as “*an approach that aims to widen intervention reach into existing systems and adheres to a predefined protocol for liner expansion and replication in other settings, which can involve scaling any number of elements to reproduce intervention effects*”. The focus is on the interaction between an intervention’s attributes and external contexts such as the implementation delivery setting (e.g., its readiness to adopt the novel approach) or the scaling environment (e.g., political climate to support investment). While it is certainly possible for system-level impact to be an objective of this approach, this is from the perspective of the role of the intervention in achieving this result, rather than starting from the perspective of the system-level outcomes and identifying what is required to achieve them.

In current global health [10], physical activity [3], and nutrition [11] scale-up frameworks, the term ‘system’ generally applies to the context or set of contexts within which an intervention takes place—e.g., the ‘health system’ or a system-level programme’—or the scale at which an intervention operates—e.g., ‘country-level system’. This use of the word contrasts with its meaning from a complex systems perspective, in which it refers to characteristics such as feedback, adaptation, and emergence. These two distinct meanings for the same word may lead to confusion, and it is important to be clear that they refer to very different concepts: one that focuses on structure, while the other is conceptual and describes non-linear complexity.

We contend that scale-up exists on a continuum: at one end, effective scaling can involve a linear, intervention-orientated expansive approach that prioritises the spread of evidence-based interventions *into* existing systems in order to drive expansion in the application of that intervention. This is the dominant, traditional model. At the other end, we contend that scale-up can sit within a complex systems paradigm in which interventions are conceptualised as ‘events in systems’ [12]. In this case, implementation and scale-up activities should focus on generating changes within the system itself to achieve the desired outcome. This we refer to as ‘systems-orientated scale-up’ to achieving population health improvement, which can complement traditional approaches in relevant situations: we propose a shift of attention from the scaling up of an intervention to achieving an outcome at system scale. We define this type of scaling as “*an approach that prioritises the behaviour and function of the system, with a focus on relations between a number of system elements, using system-level levers and dynamic system changes to drive impact at scale*”. This approach begins by considering the characteristics of the target system(s) that scaling occurs within (such as the capacity of health systems to react to change) [8] in order to identify how best to reorientate that system to achieve the desired impacts. Both approaches may make use of discrete interventions, but the part they play in scale-up may differ greatly between the two.

An example of this approach is the successful large-scale health system transformation at Denver Health in the United States of America. The system-wide implementation project identified health system capacity for innovation as a key systems-level driver for sustainable intervention implementation [13]. A core focus of the Denver Health system redesign was on the antecedent capacities of the health system (i.e., the ‘*system’s behaviour and function*’), to enable at-scale transformation of clinical and administrative processes. This included, for example, organisational capacity for implementing change and service capacity for infrastructure expansion. Understanding previous system behaviours and activities, including historical outcomes of system changes (i.e., the ‘*relations between system elements*’), led to a reduction in resistance by stakeholders [13]. Embedding project and system performance metrics enabled tracking of system-wide outcomes, which subsequently provided feedback to inform modifications to ongoing implementation (i.e., understanding how ‘*dynamic system changes affect intervention expansion, embeddedness and impact*’) [13].

Notwithstanding the potential strengths of a systems-orientated perspective, traditional intervention-orientated approaches remain highly appropriate in many circumstances; complex systems approaches are by no means universally required for scale-up. Scaled up interventions adopt many different pathways [14] and a complex problem need not always require a complex systems approach when scaling. Complexity of the problem could dictate whether a systems approach to scaling up is needed, but there are many other political, social and cultural factors that play a role in this decision-making process. This can include historical support for prevention of the problem, cultural norms regarding acceptability of the proposed evidence-based approach, and readiness of the community to adapt and integrate a ‘new way of doing things’. Complexity can relate to the properties of the intervention and the system it is being implemented into, and need not only relate to the problem or approach [15]. Likewise, population interventions may be incorporated as part of a broader, comprehensive health strategy, which may have involved prior systems analysis of the problem and target health systems change. However, the approach taken to understand the problem (systems analysis) and end goal of the strategy (systems change) are independent of the approach taken to scale relevant interventions, or achieve outcomes at scale. A population intervention can be embedded in a national strategy that adopts a systems perspective, and yet the strategy used to plan and undertake national roll-out of that intervention can remain linear.

For example, the global pandemic of coronavirus disease 2019 (COVID-19) is a complex public health problem [16], and yet responses have been scaled using both intervention- and systems-orientated approaches. For example, in Australia, public health messaging to promote social distancing was implemented nationally using a linear intervention-orientated approach. Focus was on rapidly expanding the reach and replication of the intervention strategies (e.g., signage and prompts, and infrastructure changes to control customer traffic flow) across all public spaces. Emphasis was on widening the reach of the intervention strategies into existing systems, requiring those systems to adapt in accordance to the requirements of this new ‘intervention’. In parallel, a systems-orientated approach was adopted by the federal government who introduced changes to Australia’s welfare system (e.g., introduction of a wage subsidy scheme and financial supplements) to limit the economic impact of the pandemic and attempt financial stability across sectors. Focus was on changing the welfare system structure to achieve sustainable implementation and impact of social support at scale. This example illustrates that the ‘choice’ of scale-up approach is not a dichotomous decision between ‘linear’ or ‘systems’. Decisions should be embedded in, and driven by, the complexity of the problem, context (political, social and environmental) that scaling up must occur within, the intervention, response time, costs and resources required, and capacity to impact or change the target system(s) behaviour and structure to achieve the desired outcome.

For many interventions, a linear, mechanistic scaling process allows for systematic adaptation of interventions as they move from controlled into less controlled delivery settings. It can also generate useful implementation and individual impact-related monitoring data, such as systems-level barriers and facilitators to sustainability, providing insight into prerequisites and indicators of successful scaling. Yet, some complex public health problems such as physical inactivity and obesity have remained resistant to public health intervention of this kind. They are driven by multiple determinants rooted in complex political and social systems [17, 18]. Effective, equitable, and sustainable action to tackle these kinds of problems requires changes to multiple elements across many systems, using whole-of-systems approaches [19, 20]. Although public health responses to these problems have at times incorporated systems approaches, we argue that there is all too often a disconnect between the systems thinking that underpins descriptions of the causal drivers of these complex problems and the application of systems thinking to public health action to address them.

Resources to inform the potential scalability of interventions also often frame scaling up within a ‘context-to-outcome’ conceptualisation. For example, establishing an ideal *context* for scaling up (e.g., having political buy-in) is considered as a prerequisite for achieving an ideal *outcome* at scale (e.g., increased community reach and intervention adoption). While this context-to-outcome relationship can exist, many interventions still fail to achieve state or national roll-out despite possessing these desirable ‘prerequisites’ for scale-up. A complex systems model of scaling up showed that when scaling population physical activity and nutrition interventions, there are bidirectional and dynamic relationships between scale-up contexts, mechanisms and outcomes [21]. For example, irrespective of similarities in the scale-up context (e.g., political support for the intervention), successful scale-up was dependent on the activation of different mechanisms during the implementation process [21]. Contrary to the widely accepted context-to-outcome depiction of scaling up, this research showed that by analysing scale-up using a complex systems perspective, potential leverage points to enhance future scale-up efforts could be identified. Leverage points are places within a complex system whereby a small shift in one aspect can lead to significant changes in another [22]. Failure to account for complexity when scaling up, including identifying the interaction between scale-up mechanisms and leverage points, has the potential to limit the sustainability and impact of scale-up efforts [21].

Systems change plays a major role in the relation between implementation processes and institutionalisation of interventions at scale [3, 5]. Program reach (i.e., the *spatial dimensions* of scaling up which can include location, size or proximity of adopters) has dominated the scale-up discourse and remains a major focus within public health [23]. However, the *temporal dimensions* of scaling up have largely been ignored [23]. Overemphasis on the impact of systems as merely a contextual influence on outcomes risks oversimplifying what is required to achieve population health impact, and ignores the ways in which consideration of system levers at different levels and over time may enhance outcomes. It may also implicitly reinforce the conceptualisation that scaling is a linear process and desirable rates of programme uptake (reach) are a sufficient indicator of scaling success.

## Conclusions

Complexity science and complexity-informed implementation offer promising paradigms for research–practice translation [24], but they are underutilised in scale-up models. We hypothesise that an approach to scaling that prioritises system-level actions, such as improving a system’s readiness to ‘accept’ an up-scaled intervention, in addition to intervention-orientated determinants, would provide greater insight as to whether characteristics of the system(s) would enable receptiveness to scaling in the first place. Reorientating the scale-up discourse to embrace a complex systems perspective has the potential to change the ways in which funders, policymakers and stakeholders are involved in scale-up; help researchers to generate more valuable evidence; and drive more effective planning and delivery of actions to tackle complex public health problems.

## Data Availability

Not applicable.
